# Blood‐brain barrier breakdown and abnormal brain microstructure in older adults with COVID‐19‐related cognitive impairment are modified by sex and APOE4

**DOI:** 10.1002/alz.091785

**Published:** 2025-01-09

**Authors:** Emilie T. Reas, Austin T Alderson‐Myers, Seraphina K. Solders, Qian Shen, Xin Wang, Charlotte S. Rivera, Sarah Banks, Jennifer Graves

**Affiliations:** ^1^ University of California, San Diego, La Jolla, CA USA

## Abstract

**Background:**

Persistent neurological symptoms including cognitive impairment can follow SARS‐CoV‐2 infection, a condition termed neurological post‐acute sequelae of COVID‐19 (Neuro‐PASC). Structural brain differences have been observed in individuals with Neuro‐PASC, raising concern that COVID‐19 may promote age‐related neurodegeneration. However, the mechanisms by which COVID‐19 impairs cognition and its impact on brain aging, remain poorly characterized. Blood‐brain barrier (BBB) dysfunction has been observed in Alzheimer’s disease and has been hypothesized to contribute to Neuro‐PASC, implicating potential acceleration of Alzheimer’s disease pathogenesis by COVID‐19. The present study evaluated BBB permeability and brain microstructure among older adults with Neuro‐PASC, along with modification by sex and APOE4, risk factors for Alzheimer’s disease that have also been associated with COVID‐19 outcomes.

**Method:**

Older adults (50‐90 years; 54% women) with (N=16) or without (N=43) Neuro‐PASC underwent neuropsychological testing as well as multi‐shell diffusion MRI and dynamic contrast‐enhanced MRI to measure restriction spectrum imaging (RSI) metrics of brain microstructure and BBB permeability in subcortical, white matter, and gray matter regions of interest. ANCOVA compared cognitive and MRI measures between Neuro‐PASC and healthy controls, and models were repeated assessing interactions with sex or APOE4. Partial correlations examined associations between brain microstructure and BBB permeability. Models were adjusted for age and sex, with further adjustment for education for cognitive scores, and for MRI software for RSI measures.

**Result:**

Neuro‐PASC participants had worse episodic memory performance, higher white matter BBB permeability, and lower neurite density and hindered diffusion than controls. APOE4 carriers demonstrated more pronounced PASC‐related cognitive impairment and microstructural abnormalities than non‐carriers. PASC‐related BBB leakage was stronger among men, whereas women showed stronger PASC‐related increases in free water and decreases in restricted diffusion. Among Neuro‐PASC, but not controls, hippocampal BBB permeability correlated with greater hippocampal free water.

**Conclusion:**

Older adults with persistent COVID‐19‐related neurological complaints demonstrate objective cognitive impairment accompanied by BBB dysfunction and microstructural brain abnormalities. PASC‐related cognitive decline, BBB breakdown, and microstructural injury differ by sex and are magnified among APOE4 carriers. Longitudinal investigation is warranted to determine whether PASC heightens risk for Alzheimer’s disease by accelerating cerebrovascular and neurodegenerative changes, particularly among high‐risk populations.

